# Battle of the Gases: How Air and Nitrous Oxide Affect Endotracheal Tube Cuff Pressure During General Anesthesia

**DOI:** 10.7759/cureus.67367

**Published:** 2024-08-21

**Authors:** Aparna Bagle, Abhishek Raj, Shivangi Gaur, Chandrakala Singh, Amala Kale

**Affiliations:** 1 Department of Anaesthesiology, Dr. D. Y. Patil Medical College, Hospital and Research Centre, Dr. D. Y. Patil Vidyapeeth (Deemed to be University), Pune, IND

**Keywords:** oxygen, endotracheal intubation, tracheal mucosal erosion, cuff manometer, general anaesthesia, nitrous oxide, endotracheal tube cuff pressure

## Abstract

Background

Endotracheal tube (ETT) cuff pressure changes during general anesthesia. Endotracheal cuff pressure ideally should be maintained between 20 and 30 cm of H_2_O. Cuff pressure of less than 25 cm of H_2_O increases the chances of aspiration while pressure of more than 40 cm of H_2_O causes tracheal mucosa damage. The study aimed to monitor and compare variations of endotracheal cuff pressure during general anesthesia with oxygen-air or oxygen-nitrous oxide.

Methods

This prospective, randomized, double-blinded, observational study was conducted on 40 patients. After approval from the institutional ethics subcommittee, 40 patients of either gender, aged 18-60 years, belonging to ASA grades I and II, who were undergoing elective surgery under general anesthesia, were enrolled in this study. The patients were randomly divided into two groups, with 20 in each group. In Group A, oxygen-air and Group N, oxygen-nitrous oxide was used as a gaseous mixture in general anesthesia with ETT. The ETT cuff pressure was recorded with the help of a cuff manometer at intervals of five, 10, 20, 30, 40, 50, 60, 70, 80, and 90 minutes after intubation. If pressure was more than 40 cm of H_2_O, it was reduced to 25-30 cm of H_2_O. Data were collected and analyzed using methods described in Primer of Biostatistics by Stanton A. Glantz. Quantitative data were analyzed using the Student's t-test. Qualitative data were analyzed using the chi-square test.

Results

An increase in cuff pressure was noted more in Group N as compared to Group A. The pressure in the endotracheal cuff started to gradually increase after 30-40 minutes in Group N after intubation, while in Group A, there was no significant increase. The average number of times the cuff deflated was 0.2 ± 0.41 in Group A and 1.55 ± 0.51 in Group N, which was highly significant.

Conclusion

Changes in endotracheal cuff pressure were observed when using different gas mixtures for inflation. Specifically, cuff pressure increased with oxygen and nitrous oxide compared to oxygen with air. This suggests that anesthetic gas composition can impact cuff pressure, potentially affecting tracheal mucosal perfusion and patient safety. Therefore, regular monitoring and adjustment of cuff pressure is crucial, especially when using nitrous oxide, to prevent complications and ensure optimal patient care.

## Introduction

Intubation during anesthesia is crucial for maintaining airway patency, facilitating respiration, and providing airway protection during resuscitation. It decreases breathing efforts, reduces airway dead spaces, and minimizes aspiration risks. Tracheal intubation is a fundamental step in securing the airway during general anesthesia, with cuffed endotracheal tubes (ETTs) commonly used to prevent air leaks and aspiration [1].

In the 1960s, endotracheal cuffs were made of red rubber with high-pressure-low-volume (HPLV) cuffs. Today, they are made of polyvinyl chloride (PVC) or polyurethane and are classified as high-volume-low-pressure (HVLP). Cuffs are typically inflated with air, and the standard cuff pressure of 20-30 cm H_2_O is shown to prevent air leakage and aspiration [2,3]. The risk of aspiration increases if cuff pressure falls below 25 cm H_2_O, while pressures above 40 cm H_2_O can impede capillary blood flow in the tracheal mucosal circulation, causing damage to the tracheal mucosal lining. Common complications include hoarseness, sore throat, and dysphagia. Prolonged intubation can lead to tracheal stenosis and tracheomalacia [4,5].

Endotracheal cuff pressure changes due to various factors such as the type and site of surgery and the gases used in anesthesia [6,7]. Many studies indicate that conventional methods for ETT cuff inflation and pressure measurement are unreliable, making cuff pressure monitoring with a cuff manometer the standard technique [8,9].

Nitrous oxide (N_2_O) is a weak general anesthetic commonly used as a carrier gas (mixed with oxygen) in Indian hospital settings and small nursing homes with limited availability of air as an anesthetic gas. During general anesthesia, N_2_O penetrates the semi-permeable membrane of the cuff, increasing cuff pressure over time. Cuffs made of materials like PVC are less permeable to N_2_O compared to rubber, but the increase in endotracheal cuff pressure can still cause tracheal injury [10].

This study was designed to monitor and compare variations in endotracheal cuff pressure during general anesthesia using oxygen-air versus oxygen-nitrous oxide as anesthetic gases.

## Materials and methods

The study, approved by the institutional ethics subcommittee (protocol number IESC/15/2020) and conducted in accordance with the Declaration of Helsinki, was a prospective, randomized, double-blinded observational study carried out over six months from May 2020 to October 2020. Patients were enrolled after providing written informed consent.

Based on the study by Manissery JJ et al., which compared endotracheal cuff pressure changes when using air versus nitrous oxide in anesthetic gases, the mean pressure in the nitrous group at the end of one hour was 62.60 ± 12.33 cm H_2_O, compared to 27.63 ± 3.22 cm H_2_O in the air group [11]. Using WinPepi software (Joseph H. Abramson, Jerusalem, Israel), with a significance level of 5% and a study power of 90%, the required sample size was calculated. To detect a minimum difference of 10 cm H_2_O in mean cuff pressure, a total of 36 patients (18 in each group) were required. Considering potential exclusions and dropouts, the total sample size was increased to 40, with 20 patients in each group.

The study included 40 patients of either gender, aged between 18 and 60 years, classified under American Society of Anesthesiologists (ASA) grades I and II, who were scheduled for elective surgery under general anesthesia. Patients with ASA grade III and above, a BMI greater than 30, anticipated difficult intubation, head and neck surgeries, surgeries other than in supine positions, and those for whom nitrous oxide was contraindicated were excluded from the study.

Study design

**Figure 1:**
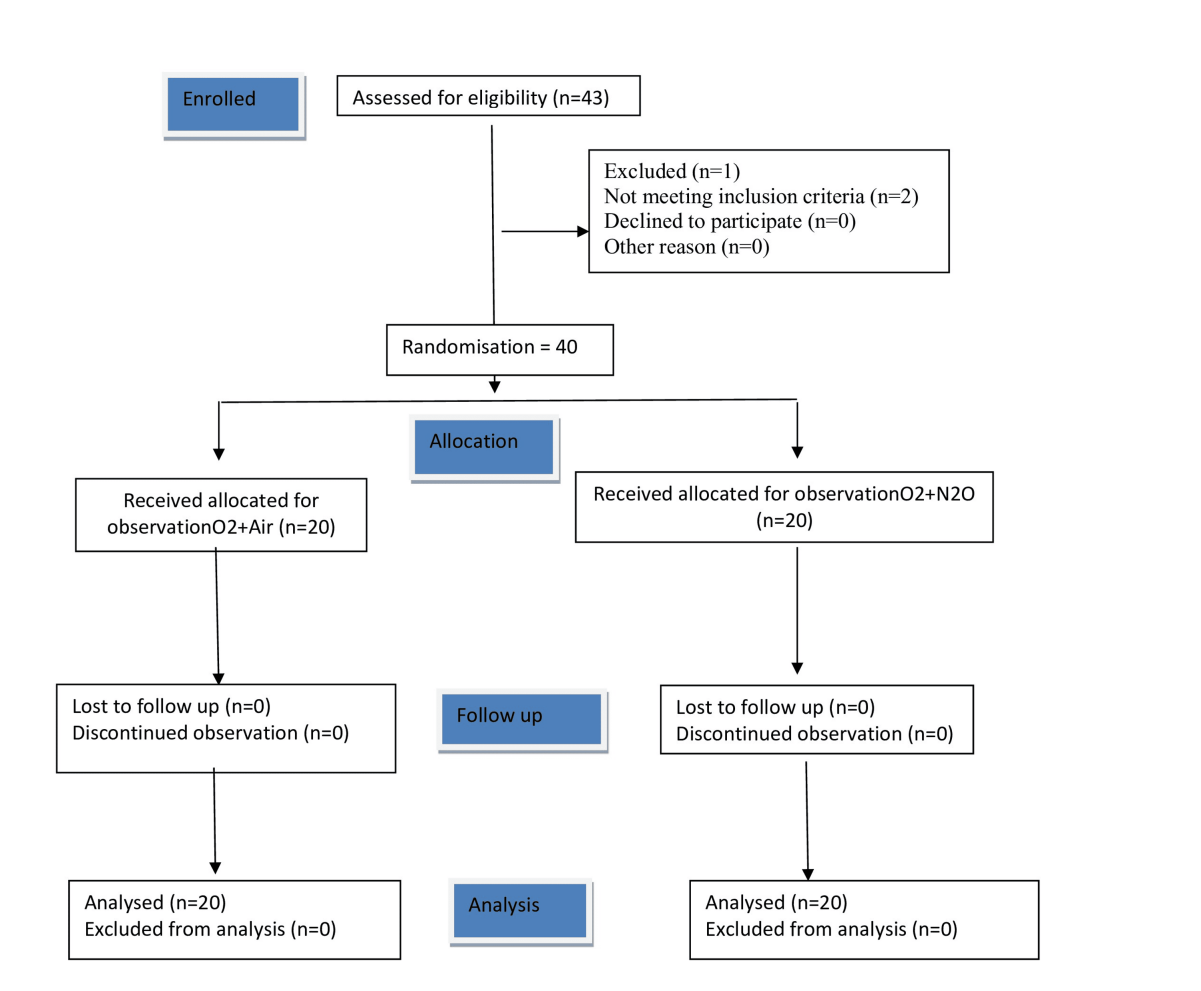
Consolidated Standards of Reporting Trials (CONSORT) statement for patient recruitment

All participants underwent a thorough pre-anesthesia evaluation, including relevant laboratory investigations, after providing informed and written consent. Patients were randomly assigned to two groups using a computer-generated random number table, with 20 patients in each group. Group A received an oxygen-air mixture, while Group N received an oxygen-nitrous oxide mixture. Both the patients and the anesthesiologist recording endotracheal cuff pressure were blinded to the group allocation. The primary anesthetist managing the case was aware of the gases being used, but the patients and the anesthesiologist measuring cuff pressure were blinded by covering the flow meter (Figure 1).

Preoperative procedure

Following confirmation of nil per oral status, standard monitors (ECG, pulse oximeter, and non-invasive blood pressure) were attached. Preoxygenation was carried out with 100% oxygen for three minutes. Premedication included glycopyrrolate (0.004 mg/kg), midazolam (0.02 mg/kg), and fentanyl (2 µg/kg).

Induction and intubation

Induction was achieved with propofol (2 mg/kg) and succinylcholine (2 mg/kg). After ensuring adequate muscular relaxation, intubation was performed using a high-volume, low-pressure PVC (Polymed) ETT. ETT sizes were seven for females and 8.5 for males. The cuff was inflated with air, and adjustments were made by palpation of the pilot balloon. A cuff pressure manometer (Mallinckrodt Medical, Athlone, Ireland) was connected, the initial reading was noted, and the cuff pressure was adjusted to 25 cm H_2_O, within the standard range of 20 to 30 cm H_2_O.

Anesthesia maintenance

Group A received an oxygen-air mixture (50:50 ratio), while Group N was administered an oxygen-nitrous oxide mixture (50:50 ratio). Anesthesia was maintained with vecuronium for muscle relaxation and isoflurane as an inhalational agent. The settings included a fresh gas flow rate of 2 L/minute, a respiratory rate of 12 breaths per minute, a tidal volume of 7 mL/kg, an inspiration/expiration (I/E) ratio of 1:2, and an end-expiratory pressure of 5 mmHg. EtCO_2_ was maintained between 32 and 38 mm Hg.

Cuff pressure monitoring

Endotracheal cuff pressure was measured and recorded at five-, 10-, 20-, 30-, 40-, 60-, 70-, 80-, and 90-minute intervals, as well as immediately before extubation, by an anesthesiologist who was blinded to the gas mixture used. If the cuff pressure exceeded 40 cm H_2_O, it was adjusted back to the initial level of 25 cm H_2_O.

Postoperative care

At the end of surgery, both groups were ventilated with 100% O_2_, and inhalation agents were discontinued. Extubation was performed after the return of spontaneous respiration and the administration of neostigmine (0.05 mg/kg) and glycopyrrolate (0.008 mg/kg). All patients received 15 mg/kg paracetamol intravenously for analgesia before extubation.

Post-anesthesia care unit (PACU) monitoring

Patients were monitored in the PACU for four hours. At one hour and 24 hours post-extubation, evaluations were made for sore throat, hoarseness, and dysphagia. All cases were completed within the stipulated time. Data were collected, tabulated, and analyzed using methods described in Primer of Biostatistics by Stanton A. Glantz. Quantitative data were analyzed using an unpaired Student's t-test, and qualitative data were assessed using the chi-square test.

## Results

The study compares two groups of participants: Group A, who received O_2_ + air, and Group N, who received O_2_ + N_2_O. Key characteristics, such as age, height, weight, gender distribution, ASA grading, and operation time, were analyzed to assess any significant differences. The p-values indicate the statistical significance of these comparisons, with more details provided in Table 1.

**Table 1:** Demographic parameters

Parameters	Group		p-value
	A (O_2_ + air)	N (O_2_ + N_2_O)	
Age (in years) (mean ± SD)	42.7 ± 8.83	43.6 ± 11.66	0.784
Height (in cm) (mean ± SD)	165 ± 7.02	167 ± 5.91	0.335
Weight (in kilograms) (mean ± SD)	58.67 ± 7.12	56.07 ± 6.472	0.234
Gender (male/female)	8/12 (40%/60%)	9/11 (45%/55%)	0.749
ASA grading (I/II)	6/14 (30%/70%)	7/13 (35%/65%)	0.735
Operation time (in minutes) (mean ± SD)	113.69 ± 72.43	119.23 ± 66.79	0.802

The above data show no statistically significant differences in terms of age, height, weight, operation time, gender, and ASA grading (p > 0.05).

Initially, in both the groups, i.e., Group A and Group N, there was no significant difference seen in cuff pressure changes for the first 20 minutes (p > 0.05). Later on, it was observed that in Group N, cuff pressure started to increase significantly throughout the general anesthesia, and sometimes it crossed 40 cm of water and needed to release pressure, whereas in Group A, the cuff pressure remained more or less constant. Table 2 presents the comparison of measurements between Group A (O_2_ + air) and Group N (O_2_ + N_2_O) at various time intervals. At five and 10 minutes, the groups show no significant difference (p = 0.432 and p = 0.220, respectively). However, from 30 minutes onward, Group N consistently exhibits higher values compared to Group A, with significant differences (p = 0.001) observed at 30, 40, 50, 60, 70, 80, and 90 minutes and pre-extubation.

**Table 2:** Endotracheal tube cuff pressure changes in Group A and Group N *Statistically significant p-value < 0.05

Time interval	Cuff pressure of Group A (O_2_ + air)	Cuff pressure of Group N (O_2_ + N_2_O)	p-value
Five minutes	26.78 ±2.38	27.35 ± 2.15	0.432
10 minutes	27.15 ± 2.56	28.27 ± 3.1	0.220
20 minutes	33.27 ± 3.17	34.79 ± 3.89	0.184
30 minutes	34.78 ± 4.17	42.54 ± 5.12	0.001*
40 minutes	33.15 ± 4.76	40.54 ± 5.34	0.001*
50 minutes	31.75± 5.78	42.79 ± 7.79	0.001*
60 minutes	32.79 ± 6.69	44.16 ± 6.67	0.001*
70 minutes	30.76 ± 5.59	36.17 ± 7.16	0.001*
80 minutes	31.77 ± 6.79	40.15 ± 6.01	0.001*
90 minutes	32.03 ± 5.63	42.17 ± 5.63	0.001*
Pre-extubation	30.05 ± 3.42	40.78 ± 5.52	0.001*

In Group N, significantly more number of times, intra-tracheal cuff pressure exceeds 40 mmHg and needed to deflate (Table 3).

**Table 3:** Comparison of average endotracheal tube cuff deflations in Groups A and N over 90 minutes *Statistically significant p-value < 0.05

	Group A (O_2_ + air)	Group N (O_2_ + N_2_O)	p-value
Number of deflations	0.2 ± 0.41	1.55 ± 0.51	0.001*

The incidence of sore throat and hoarseness at two and 24 hours post-procedure between Group A and Group N is compared in Table 4. Postoperatively, after two hours, nine patients in Group A and 11 patients in Group N experienced sore throats. Similarly, four patients in Group A and three patients in Group N experienced sore throat at 24 hours, which was statistically insignificant.

**Table 4:** Incidence of postoperative sore throat and hoarseness in Group A and Group N

	Group A	Group N	p-value
Sore throat			
At two hours	9 (45%)	11 (55%)	0.750
At 24 hours	4 (20 %)	3 (15%)	1.000
Hoarseness			
At two hours	3 (15%)	2 (10%)	1.000
At 24 hours	0 (0%)	1 (5%)	1.000

## Discussion

Endotracheal cuffs are currently made of PVC material, which is permeable to nitrous oxide but less so than red rubber cuffs. Nitrous oxide is 35 times more soluble in blood than nitrogen, allowing it to rapidly diffuse into a cuff filled with air. In a study by Manissery JJ et al., ETT cuff pressures were compared during general anesthesia using air versus a 50% mixture of nitrous oxide and oxygen as inflating agents [11]. The study evaluated the effectiveness of using a 50% mixture of nitrous oxide and oxygen (50% N_2_O) to maintain stable cuff pressures during general anesthesia with 67% N_2_O. The mean cuff pressure with air (control) as the inflating agent was 62.60 ± 12.33 cm H_2_O at the end of one hour of general anesthesia, while the mean pressure with 50% N_2_O was 27.63 ± 3.22 cm H_2_O. This indicates that using a 50% N_2_O ratio rather than air maintains more stable intra-cuff pressure. Diffusion typically starts 30 to 40 minutes after airway instrumentation, causing cuff pressure to increase, which can lead to tracheal vessel obstruction and mucosal damage. Tracheal mucosa microcirculation obstruction occurs at 30 cm H_2_O, with ischemic changes observed at 45 cm H_2_O [12]. Several factors affect ETT cuff pressure, including age, gender, body mass index, ETT size, type of surgery, patient position, and type of ventilation [13,14]. Table 1 shows no statistically significant differences in demographic values (age, gender, operation time, ASA grading) between groups (p > 0.05). A cuff manometer was used to detect initial cuff pressure just after intubation and monitor changes throughout general anesthesia.

From Table 2, it is evident that there was no statistically significant difference in cuff pressure change for the initial 30 minutes of general anesthesia between Groups A and N. After 30 minutes, a significant rise in cuff pressure was observed in Group N compared to Group A (p < 0.05). The cuff pressure continued to increase in Group N until pre-extubation, necessitating regular reductions with cuff manometer monitoring.

In a study by Mogal SS et al., comparing ETT cuff pressure changes using air versus nitrous oxide in anesthetic gases during laparoscopic abdominal surgeries, the increase in cuff pressure in Group N was greater at all time points studied (p < 0.001). In Group N, cuff pressure increased significantly every 30 minutes throughout the procedure (p < 0.05), indicating a higher rising trend than in Group A [15].

In the study by Karasawa F et al., laparoscopy induced an increase in cuff pressure in both study groups, but the use of N_2_O in Group N led to an excessive rise in cuff pressure and a significant difference between the two groups. Although our study did not include cuff pressure changes in laparoscopic surgeries, the use of nitrous led to significant cuff pressure changes [16].

The study by Puthenveettil N et al. showed that mean intra-cuff pressure increased significantly in the air group (start 14 mmHg, end 40.9 mmHg), while the saline group showed no significant increase (start 12.7 mmHg, end 14.6 mmHg). Using saline reliably resulted in sustained low intra-cuff pressures [17].

Koşar O et al. concluded that cuff pressure values were significantly higher (p < 0.001) in the N_2_O group throughout anesthesia compared to the air group. The N_2_O group also had a higher incidence of sore throat and hoarseness [18]. From Graph 2, 11 (55%) of 20 patients in Group N reported a postoperative sore throat, compared to nine (45%) in Group A, showing no significant statistical difference (p > 0.05). Table 4 indicates comparable incidence rates of hoarseness and sore throat between groups, as cuff pressure was maintained below 45 cm H_2_O and regularly monitored. Our findings were similar to the observation by Benneth MH that cuff inflation guided by a manometer significantly reduces the incidence of postoperative sore throats [19].

Monitoring and comparing variations in endotracheal cuff pressure during general anesthesia with oxygen-air versus oxygen-nitrous oxide is crucial for patient safety, optimizing anesthesia, and informing economic considerations, clinical guidelines, and technological advancements. Such studies help ensure adequate ventilation and minimize complications associated with cuff pressure fluctuations, thereby improving postoperative outcomes.

However, there are limitations to consider. The study did not account for variations in patient positions (such as prone, head-low, or lateral), which could affect cuff pressure; it did not compare the effects of different inhalational agents on cuff-pressure changes; the small sample size may restrict the generalizability of the findings; and the impact of cuff-pressure changes in laparoscopic versus non-laparoscopic surgeries was not investigated.

## Conclusions

Changes in endotracheal cuff pressure were observed when using different gas mixtures for inflation. Specifically, cuff pressure increased with oxygen and nitrous oxide compared to oxygen with air. This suggests that anesthetic gas composition can impact cuff pressure, potentially affecting tracheal mucosal perfusion and patient safety.

Our study compared endotracheal cuff pressure changes when using nitrous oxide with oxygen versus nitrous oxide with air. We found that cuff pressure increased significantly when using nitrous oxide with oxygen but remained relatively stable with nitrous oxide and air.
